# White Spot Syndrome Virus-Induced Shrimp miR-315 Attenuates Prophenoloxidase Activation via *PPAE3* Gene Suppression

**DOI:** 10.3389/fimmu.2018.02184

**Published:** 2018-09-25

**Authors:** Phattarunda Jaree, Chantaka Wongdontri, Kunlaya Somboonwiwat

**Affiliations:** ^1^Center of Excellence for Molecular Biology and Genomics of Shrimp, Department of Biochemistry, Faculty of Science, Chulalongkorn University, Bangkok, Thailand; ^2^Omics Sciences and Bioinformatics Center, Faculty of Science, Chulalongkorn University, Bangkok, Thailand

**Keywords:** microRNA, viral infection, invertebrates, *Penaeus monodon*, prophenoloxidase

## Abstract

MicroRNAs (miRNAs), the small non-coding RNAs, play a pivotal role in post-transcriptional gene regulation in various cellular processes. However, the miRNA function in shrimp antiviral response is not clearly understood. This research aims to uncover the function of pmo-miR-315, a white spot syndrome virus (WSSV)-responsive miRNAs identified from *Penaeus monodon* hemocytes during WSSV infection. The expression of the predicted pmo-miR-315 target mRNA, a novel *PmPPAE* gene called *PmPPAE3*, was negatively correlated with that of the pmo-miR-315. Furthermore, the luciferase assay indicated that the pmo-miR-315 directly interacted with the target site in *PmPPAE3* suggesting the regulatory role of pmo-miR-315 on *PmPPAE3* gene expression. Introducing the pmo-miR-315 into the WSSV-infected shrimp caused the reduction of the *PmPPAE3* transcript level and, hence, the PO activity activated by the *Pm*PPAE3 whereas the WSSV copy number in the shrimp hemocytes was increased. Taken together, our findings state a crucial role of pmo-miR-315 in attenuating proPO activation via *PPAE3* gene suppression and facilitating the WSSV propagation in shrimp WSSV infection.

## Introduction

MicroRNAs (miRNAs) are small non-coding RNA molecules transcribed from the genome and subsequently processed by Drosha and Dicer nucleases (1). Generally, when miRNA complementary binds to the 3′-untranslated regions (3′-UTRs) of mRNAs, it can cleave the target mRNAs or inhibits the mRNA translation, thus, down-regulating the corresponding gene expression. In addition, it has been reported that the miRNAs can also bind to the 5′-untranslated regions (5′-UTR) (2) or even within the coding sequence of mRNAs (3). Accordingly, miRNA's function involves the regulation of various biological processes including the host-virus interaction upon viral infection. During virus infection, viral miRNAs downregulate specific viral and cellular mRNAs to establish a host environment conducive to the completion of viral life cycle. At the same time, the host miRNAs modulate both cellular and viral transcripts exerting influence over immune responses (4).

In crustaceans, there are many reports showing that the host miRNAs can regulate the expression of the host and viral genes and vice versa. In 2012, the first large-scale miRNA characterization in white spot syndrome virus (WSSV)-infected *Marsupenaeus japonicas* lymphoid organ has been reported (5). Several cellular WSSV-responsive miRNAs play important roles in shrimp immunity including apoptosis pathway (6, 7), phagocytosis (8), NF-kB pathway (9), and JAK/STAT pathway (10). Furthermore, the WSSV miRNAs were also identified and probably could target either the host or viral genes. In the WSSV-infected *M. japonicas*, the viral miRNAs could target virus transcripts and further promote virus infection (11). In the meantime, a viral miRNA, WSSV-miR-N24, could target the shrimp apoptotic gene, caspase 8, and further represses the apoptosis of shrimp hemocytes (12). Despite this, the study on the host miRNAs-virus relationship in shrimp is still very limited.

In *Penaeus monodon*, the 60 known miRNA homologs that are expressed in the hemocytes of WSSV-challenged shrimp at the early and late phases of infection have been identified (13). Their immune-related gene targets in apoptosis pathway, antimicrobial peptide, prophenoloxidase system, signal transduction, proteinase and proteinase inhibitor, blood clotting system, and heat shock protein have also been predicted. Among them, the pmo-miR-315 was found to be a highly up-regulated miRNA in response to WSSV challenge and its target gene was predicted to be a novel prophenoloxidase-activating enzyme, called *PmPPAE3* (13). According to the previous reports, the miR-315 was also up-regulated after WSSV infection in lymphoid organ of *M. japonicas* (5) and hepatopancreas of *F. chinensis* (14). However, the function of miR-315 in shrimp immunity against WSSV infection is still unknown. In this research, the cellular mRNA target of pmo-miR-315 was confirmed. Moreover, the role of pmo-miR-315 in WSSV-infected shrimp was investigated.

## Materials and methods

### Shrimp and WSSV

Healthy black tiger shrimp, *P. monodon*, of about 3–5 and 15–20 g in size were purchased from a farm in Surat Thani Province, Thailand. Due to the Ethical Principles and Guidelines for the use of animals for scientific purposes by the National Research Council of Thailand, this project was conducted according to the animal use protocol number 1823006 approved by the Chulalongkorn University Animal Care And Use Committee (CU-ACUC). The animals were reared in laboratory tanks at ambient temperature, and maintained in aerated water with a salinity of 15 ppt for at least 7 days before use.

The WSSV stock used for the experimental infection was prepared according to the method described by Xie et al. (15) and stored at −80°C until used. One microliter of WSSV stock was 10-fold serially diluted with 0.85% NaCl to 10^−7^ dilution. The diluted WSSV of 50 μL was injected into the second abdominal segment of the shrimp. The mortality was recorded daily. This dosage caused 100% mortality within 3–4 days after injection and was used for all subsequent viral infection experiment.

This project has been reviewed and approved by the CU-IBC (Approval number: SCI-01-001) to be in accordance with the levels of risk in pathogens and animal toxins listed in the Risk Group of Pathogen and Animal Toxin (2017) published by the Department of Medical Sciences, Ministry of Public Health, the Pathogen and Animal Toxin Act (2015) and Biosafety Guidelines for Modern Biotechnology, BIOTEC (2016).

### Pmo-miR-315 mimic and its scramble miRNA

The mimic microRNAs used in this research were synthesized by a commercial service GenePharma (Shanghai). The sequences of mimic pmo-miR-315 and pmo-miR-315 scramble were 5′-UUUUGAUUGUUGCUCAGAAG-3′ and 5′-GUGGUUAGCGUUAAUUCUAU-3′, respectively. The sequences of miR-315 inhibitor (AMO-miR-315) and miR-315 inhibitor scramble were 5′-CUUCUGAGCAACAAUCAAAA-3′ and 5′-ACGAACCUACGAUAAUAAUC-3′.

### Pmo-miR-315 expression in WSSV-infected shrimp tissues

The expression of pmo-miR-315 in various shrimp tissues including hemocytes, gill, lymphoid organ and stomach of WSSV-infected *P. monodon* was determined by stem-loop quantitative real time RT-PCR (qRT-PCR). The pooled total RNA of each tissue from 3 WSSV-infected individuals at 0, 6, 24, and 48 hpi was prepared using mirVana miRNA Isolation Kit (Life Technologies) and 1 μg was used as templates for the first strand stem-loop cDNA synthesis using Superscript III Reverse Transcriptase (Invitrogen). The qPCR was performed using SsoFast™ EvaGreen® Supermix (Bio-Rad) on CFX96 touch real-time PCR detection system (Bio-Rad), where each sample was analyzed in triplicate. The amplification condition was 98°C for 2 min, followed by 40 cycles of 95°C for 5 s and 60°C for 30 s. A non-coding small nuclear RNA, U6, was used as a reference. The primer sequences for stem-loop cDNA synthesis, pmo-miR-315: RT-pmo-miR315_F and RT-pmo-miR315_R, and for U6: RT-U6-F and RT-U6-R, are shown in Table 1. The miRNA relative expression level was calculated using the equation by Pfaffl (16).

**Table 1:** List of primers used in this study.

**Primer name**	**Sequence (5′-3′)**
Stem-loop pmo-miR-315	GTCGTATCCAGTGCAGGGTCCGAGGTATTCGCAC TGGATACGACCTTCTG
RT-pmo-miR315_F	CGGGCGTTTTGATTGTTGCTCAG
RT-pmo-miR315_R	CCAGTGCAGGGTCCGAGGTA
RT-U6-F	CTCGCTTCGGCAGCACA
RT-U6-R	AACGCTTCACGAATTTGCGT
RT-*Pm*PPAE3-F	GGACGAGTGCCAGTTCAACA
RT-*Pm*PPAE3-R	GGTCGTTGTGGTGGTGGTCACT
RT-EF1α-F	GGTGCTGGACAAGCTGAAGGC
RT-EF1α-R	CGTTCCGGTGATCATGTTCTTGATG
knPPAE3-F	CAACATTGCCGGACTGCCTA
knPPAE3-R	GGCAGAAGCACGACACGAAC
knPPAE3-T7-F	TAATACGACTCACTATAGGCAACATTGCCGGACTG CCTA
knPPAE3-T7-R	TAATACGACTCACTATAGGGGCAGAAGCACGACA CGAAC
knGFP-F	ATGGTGAGCAAGGGGGAGGA
knGFP-R	TTACTTGTACAGCTCGTCCA
knGFP-T7-F	GGATCCTAATACGACTCACTATAGGATGGTGAGCA AGGGGGAGGA
knGFP-T7-R	GGATCCTAATACGACTCACTATAGGTTACTTGTACA GCTCGTCCA
pmirG_*Pm*PPAE3_F	CTAGCGAGCTCCCAACGACCAGTAGGCCTGTGA
pmirG_*Pm*PPAE3_R	CTAGCTCTAGAGGCAGAAGCACGACACGAA
VP28-140Fw	AGGTGTGGAACAACACATCAAG
VP28-140Rv	TGCCAACTTCATCCTCATCA
pri-*Pm*PPAE3	GGTCGTTGTGGTGGTGGTCACT
nested *Pm*PPAE3	TCCTGACATCCTCCGTTGTTGCTCAC

In addition, the expression of the target mRNA of pmo-miR-315, a putative prophenoloxidase-activating enzyme (*Pm*PPAE3), was determined in hemocytes of shrimp infected with WSSV at 0, 6, 24, and 48 hpi. The total RNA from hemocytes of 3 individuals was reverse-transcribed into the first strand cDNA using oligo (dT) as a primer following the manufacturer's instruction for RevertAid First Strand cDNA Synthesis Kit (Thermo scientific). Quantitative real-time PCR was performed as described previously using *EF-1*α gene as an internal control.

### Specificity and inhibitory activity of pmo-miR-315 on target *pmPPAE3* mRNA

First, the pmirGLO vector (Promega) harboring the 200 bp *Pm*PPAE3 gene fragment that contained the pmo-miR-315 binding region (Figure 2A) was constructed. The *PmPPAE3* gene fragment was PCR amplified from *P. monodon* hemocyte cDNAs using the gene specific primers, pmirG_*Pm*PPAE_F and pmirG_*Pm*PPAE_R (Table 1), digested with *Sac*I and *Xba*I (New England Biolabs), cloned into the likewise double digested pmirGLO, and transformed into an *Escherichia coli* stain XL1-blue. The recombinant plasmid, pmir-T315, was extracted and sequenced to confirm the correctness of the sequences (Macrogen, Korea).

To investigate the inhibitory activity of pmo-miR-315 on the *PmPPAE3* gene target sequence, dual-luciferase activity assay was performed. The HEK293T cell culture of 8 × 10^4^ cells/well was seeded into a 24-well plate. At 24 h after seeding, 200 ng of pmir-T315 and 20 pmole of mimic miRNA (pmo-miR-315 or scramble) were co-transfected into the HEK293T cells using the Effectene® transfection reagent (Qiagen). The luciferase activity was measured using Dual-luciferase® Reporter Assay System (Promega) at 48 h post-transfection. The control cells were transfected with pmir-T315 alone and co-transfected pmir-T315 with mimic miRNA scramble.

### Introducing and silencing of pmo-miR-315 in WSSV-infected shrimp

To confirm the inhibitory effect of pmo-miR-315 on the *PmPPAE3* target gene expression in shrimp after WSSV infection, the exogenous pmo-miR-315 mimic and anti-pmo-miR-315 (AMO-miR-315) were introduced into the WSSV-infected shrimp and the expression levels of pmo-miR-315 and target gene were quantified.

To study the effect of pmo-miR-315 *in vivo*, the exogenous pmo-miR-315 was introduced into the shrimp by injection. For pmo-miR-315 silencing, the AMO-miR-315 was injected into the shrimp. In these experiments, shrimp were divided into five groups of three individuals each. The first group was injected with 0.85% NaCl used as an injection control. The miRNA and AMO control groups were shrimp injected with scramble miRNA or scramble AMO-miR-315, respectively. The two test groups were shrimp injected with pmo-miR-315 mimic or AMO-miR-315. At 2 h after the first injection, all groups were muscularly injected with WSSV. After 48 h post-WSSV infection, shrimp hemolymph was collected. The total RNA was extracted and used for the first-strand cDNA production. The expression level of pmo-miR-315 and putative *PmPPAE3* was determined by qRT-PCR.

### Analysis of WSSV copy number using qRT-PCR

To study the effect of pmo-miR-315 on WSSV replication, the WSSV copy number was also investigated according to a method described by Mendoza-Cano and Sánchez-Paz (17). The genomic DNA of WSSV-infected shrimp hemocytes was extracted using a FavorPrep™ Tissue Genomic DNA Extraction Mini Kit (Favorgen). The quantity and quality of genomic DNA was determined by NanoDrop 2000c spectrophotometer (Thermo Sciencetific) and 1.2% agarose gel electrophoresis.

The qRT-PCR was performed in triplicates of 10 μL reaction containing 5 μL Luna® Universal qPCR Master mix (New England Biolabs) and 1.5 μL genomic DNA template (10 ng/ μL) and 250 nM VP28-140Fw and VP28-140Rv (Table 1). The amplification condition was 98°C for 2 min, followed by 40 cycles of 95°C for 5 s and 61°C for 30 s. The plasmid containing a highly conserved region of the WSSV *VP28* gene, was used to generate a standard curve for the determination of WSSV copy number.

### Phenoloxidase activity assay

The phenoloxidase (PO) activity was determined in the hemolymph of WSSV- infected shrimp. The shrimp hemolymph was collected at 48 h post-WSSV infection and the PO activity was measured using a method modified from Sutthangkul et al. (18). Briefly, 50 μL of hemolymph was mixed with 25 μL of 3 mg/mL freshly prepared L-3, 4-dihydroxyphenylalanine (L-DOPA; Fluka) and 25 μL of 20 mM Tris-HCl pH 8.0. The absorbance at 490 nm was monitored. The amount of hemolymph proteins was measured by the Bradford method (19). The PO activity was recorded as A_490_/mg total protein/min.

### Identification of full-length cDNA of putative *pmPPAE3*

According to our previous report on pmo-miR-315 target prediction (13), the partial nucleotide sequence of *PmPPAE3* was obtained from *P. monodon* EST database. To identify the full-length cDNA, the 5′-RACE was performed using a SMARTer™ RACE cDNA Amplification Kit (Clontech, USA) according to the manufacturer's instructions. The specific primers of partial putative *PmPPAE3* gene were designed, which are pri-*Pm*PPAE3 and nested *Pm*PPAE3 (Table 1). The primary and nested-PCR were used to amplify the 5′-RACE cDNA library using Advantage® 2 polymerase mix (Clontech). The PCR product was analyzed by 1.2% agarose gel electrophoresis. The nested PCR product was purified and cloned into the pGEM-T easy vector (Promega) and transformed into *E. coli* XL1-blue. The positive clone was selected and sequenced by a commercial service (Macrogen, Korea). The 5′-RACE *PmPPAE3* nucleotide sequence was analyzed and combined with the starting *PmPPAE3* sequence from the EST library.

The full-length cDNA of putative *PmPPAE3* was analyzed using a Blast program (http://www.ncbi.nlm.nih.gov/BLAST/). The open reading frame (ORF) and the encoded amino acid sequence were predicted using ExPASy software (http://web.expasy.org/translate/). The putative signal peptide cleavage site was predicted by SignalP 4.1 server (http://www.cbs.dtu.dk/services/SignalP/). Moreover, the structural protein domains were analyzed by a simple modular architecture research tool program (SMART) (http://smart.embl-heidelberg.de/).

Multiple amino acid sequence alignment was performed using the Clustal Omega program (http://www.ebi.ac.uk//Tools/msa/clustalo/). The deduced amino acid sequence of *Pm*PPAE3 was aligned with other insect and crustacean PPAEs including *Bombyx mori* PO-activating enzyme (*Bm*PAE: NP_001036832.1); *Manduca sexta* proPO-activating proteinase 1 (*Ms*PAP1: AF059728), *Holotrichia diomphalia* proPO-activating factor I (*Hd*PPAFI: AB013088), *Glossina morsitans* (*Gm*PPAE: ABC84592) and *Drosophila melanogaster* melanization protease1 (*Dm*MP1: NM141193); *P. monodon* proPO-activating enzyme 1, 2, and 3 (*Pm*PPAE1: FJ595215, *Pm*PPAE2: FJ620685, *Pm*PPAE3: MH325330), *Litopenaeus vannamei* proPO-activating enzyme (*Lv*PPAE: AFW98991.1), *Fenneropenaeus chinensis* proPO-activating enzyme (*Fc*PPAE: AFW98985.1) and *Pacifastacus leniusculus* proPO-activating enzyme (*Pl*PPAE: AJ007668). An unrooted phylogenetic tree was constructed by the neighbor-joining method based on the amino acid sequences using MEGA 7 software (20). The bootstrap sampling was reiterated 1000 times.

### Tissue distribution analysis of *pmPPAE3* gene

The *PmPPAE3* gene expression in various tissues including hemocytes, gill, lymphoid organ, and stomach of healthy unchallenged *P. monodon* was analyzed by semi-quantitative RT-PCR. The total RNA of each tissue was extracted by Tri reagent (Geneaid) and used for the first-strand cDNA synthesis using RevertAid First Strand cDNA Synthesis Kit (Thermo Scientific). The PCR reaction was performed using the *PmPPAE3* specific primer pair (Table 1) and the *EF-1*α was used as an internal control. The expression profile was analyzed by 1.5% agarose gel electrophoresis.

### Effect of *pmPPAE3* gene silencing on PO activity in shrimp

The function of *Pm*PPAE3 in shrimp was characterized using a RNA interference technique. The *PmPPAE3* DNA template was amplified from normal shrimp cDNA with gene specific primers (knPPAE3-F, knPPAE3-R, knPPAE3-T7-F, and knPPAE3-T7-R) as shown in Table 1. In addition, the dsRNA of the green fluorescent protein (*GFP*) as the negative control, was prepared from the pEGFP-1 vector (Clontech) using the knGFP-F, knGFP-R, knGFP-T7-F, and knGFP-T7-R (Table 1). The *PmPPAE3*-dsRNA and GFP-dsRNA were prepared using T7 RiboMAX™ Express Large Scale RNA Production System (Promega) according to the manufacturer's instruction.

The *P. monodon* of approximately 3 g body weight were divided into two groups of three individuals each. The first (control) group was injected with 5 μg/g shrimp of *GFP*-dsRNA, whilst the second group, the *PmPPAE3* knockdown, was injected with 5 μg/g shrimp *PmPPAE3*-dsRNA. The hemolymph of individual shrimp was collected at 48 h post-injection. Then, the expression level of *PmPPAE3* gene and also the PO activity were measured as described above.

### Statistical analysis

All experiments were performed in triplicate. One-way ANOVA with Duncan's multiple range test was used to identify statistically significant differences with SPSS software (version 17.0). Data are presented as means ± 1 standard deviations of three biological replications. The statistical significance of differences among means was calculated by the paired-samples *t*-test where the significance was accepted at the *P*-value < 0.05.

## Results

### Expression of pmo-miR-315 in tissues of WSSV-infected shrimp

**Figure 1:**
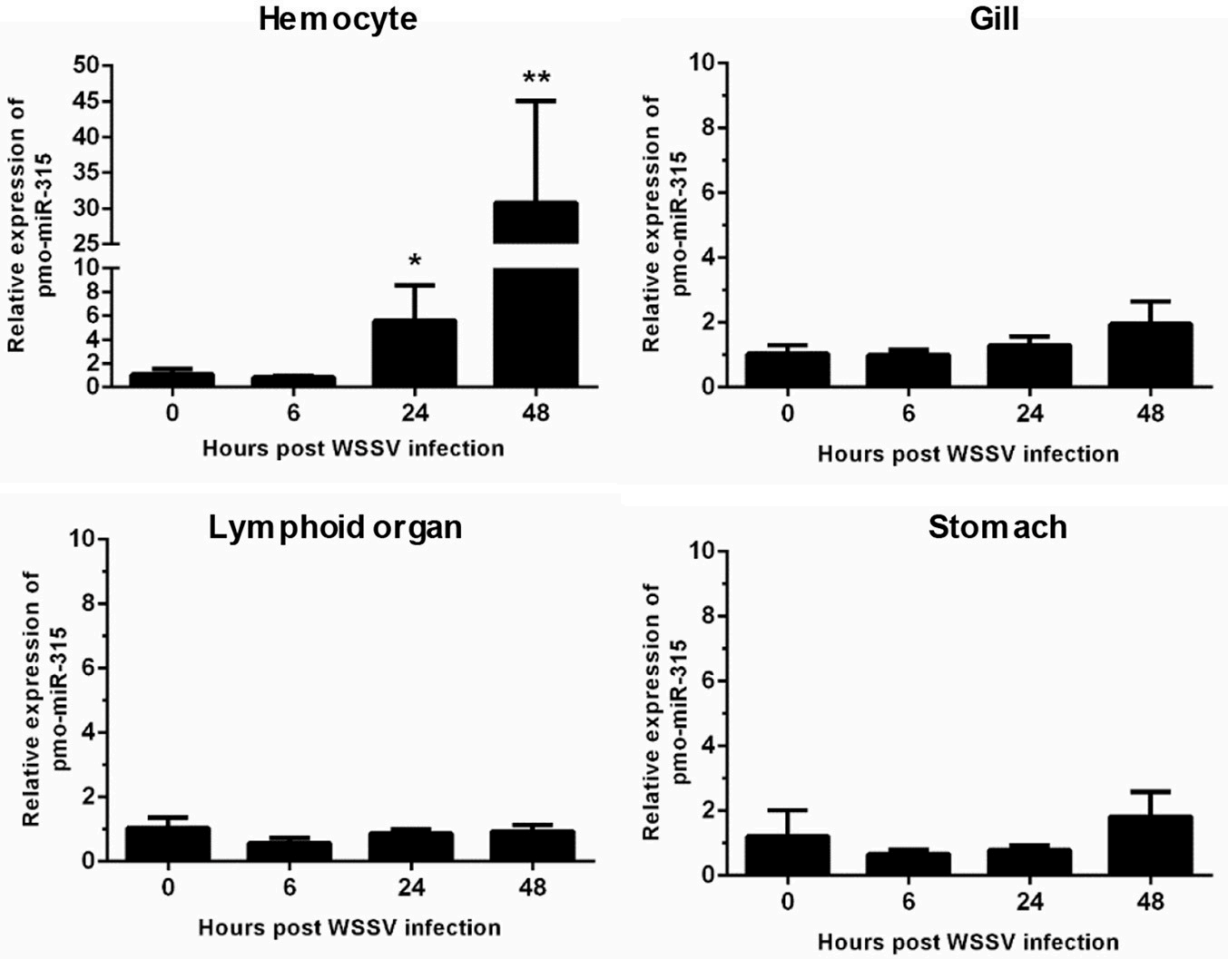
The expression of pmo-miR-315 in WSSV-infected shrimp tissues. Quantitative stem-loop real-time RT-PCR was performed to determine the expression level of pmo-miR-315 in hemocytes, gill, lymphoid organ and stomach at 0, 6, 24, and 48 hpi. Using U6 as an internal control, the relative expression level of miR-315 was calculated. All experiments were performed in triplicate. The ^*^ and ^**^ indicate the significant difference at *P* < 0.05 and *P* < 0.01, respectively.

Previously, the differentially expressed miRNAs in hemocytes of WSSV-infected shrimp were identified (13). Among them, the pmo-miR-315 was highly up-regulated at 48 h post-WSSV infection. The pmo-miR-315 expression level in various tissues including hemocytes, gill, lymphoid organ, and stomach in WSSV-infected shrimp at 0, 6, 24, and 48 hpi was further studied. Although, the pmo-miR-315 expression was observed in all tissues tested, the significant response to WSSV infection were found only in hemocytes, in which it was up-regulated at 24 and 48 hpi for about 5- and 30-fold compared with that at 0 hpi (Figure 1). Our results suggested that the pmo-miR-315 was the WSSV-responsive miRNA that might have a function in shrimp immune response.

### Expression of pmo-miR-315 target gene in WSSV-infected shrimp hemocytes

**Figure 2:**
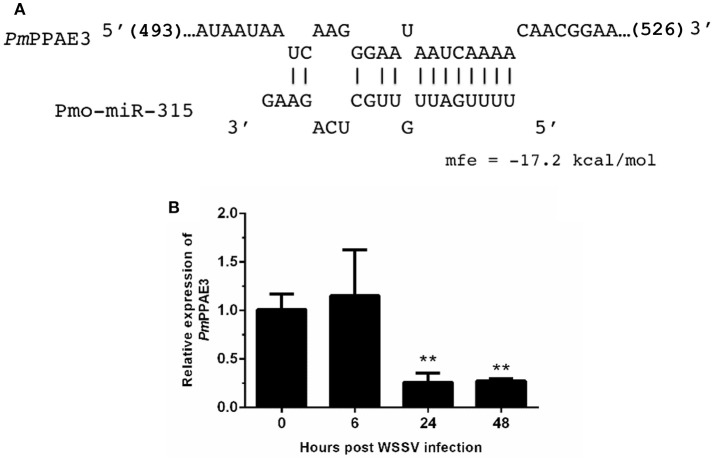
The pmo-miR-315 target mRNA, prophenoloxidase-activating enzyme 3 (*PmPPAE3*). **(A)** The pmo-miR-315/*PmPPAE3* base pairing and the binding energy was predicted using RNA hybrid software. The number in bracket indicates the position of nucleotide of pmo-miR-315 target site on the *PmPPAE3* CDS. **(B)** The relative expression level of *PmPPAE3* gene at 0, 6, 24, and 48 h post-WSSV infection was investigated. The *EF-1*α gene was used as an internal control. All experiments were performed in triplicate. The ^**^ indicates the significant difference at *P* < 0.05.

As stated before, the target mRNA of pmo-miR-315 has been predicted as a putative prophenoloxidase-activating enzyme (*PmPPAE*) by bioinformatic approaches (13). The PPAE is known as an enzyme that converts the prophenoloxidase (proPO) to active phenoloxidase (PO) in cascades of the proPO system (21). Previously, two isoforms of PPAEs including *Pm*PPAE1 and *Pm*PPAE2 were identified in *P. monodon* (22, 23). Preliminary analysis showed that the sequence of target mRNA of pmo-miR-315 was a novel isoform of *PmPPAE*. Herein, therefore, it was named as *PmPPAE3*. The RNA hybrid software used to analyze the structure and stability of miRNA/mRNA duplex showed that the seed region of pmo-miR-315 was perfectly complementary to the target sequence located on the coding sequence of *PmPPAE3* mRNA (Figure 2A).

The possibility of *PmPPAE3* transcript on being the pmo-miR-315 target was evaluated by the determination of the pattern of *PmPPAE3* gene expression in comparison to that of pmo-miR-315. The expression of *PmPPAE3* transcript was dramatically decreased by about 5-fold at 24 and 48 hpi compared with that at 0 hpi (Figure 2B). The negative correlation of expression pattern of pmo-miR-315 (Figure 1A) and *Pm*PPAE3 (Figure 2B) during WSSV infection was noted, suggesting that the pmo-miR-315 was likely to inhibit the *PmPPAE3* expression.

### Interaction between pmo-miR-315 and *pmPPAE3 in vitro*

**Figure 3:**
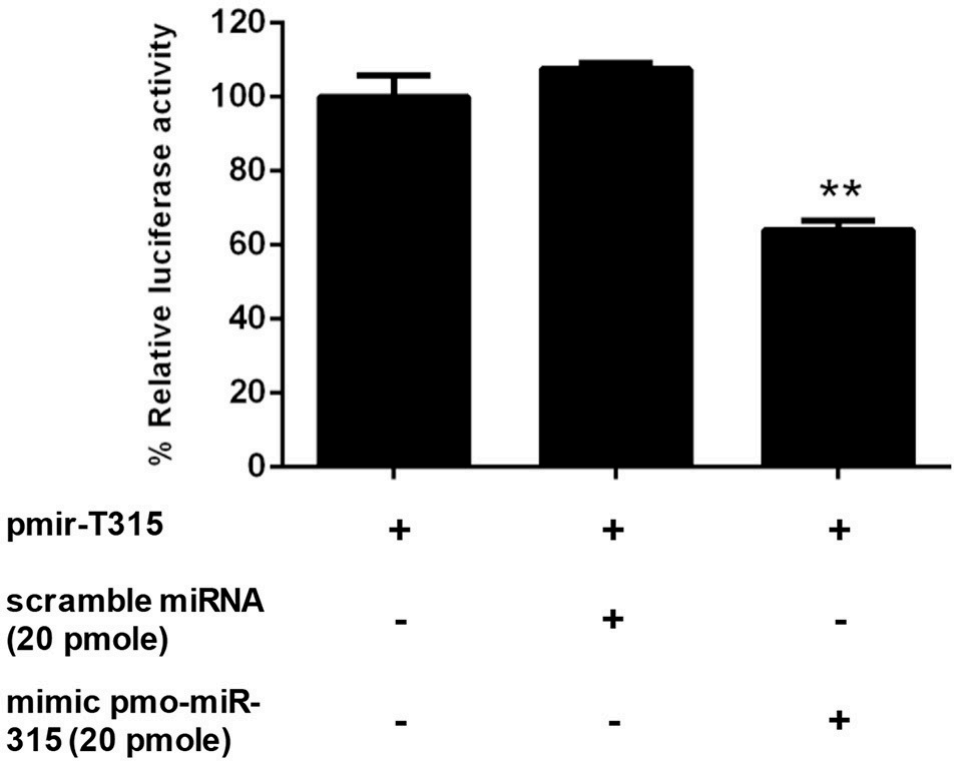
The pmo-miR-315/*PmPPAE3* interaction by luciferase reporter assay. The target sequence of pmo-miR-315 from *PmPPAE3* was amplified and cloned into the pmirGLO vector (pmir-T315). The pmo-miR-315 mimic or scramble pmo-miR-315 were co-transfected with pmir-T315 using Effectene transfection reagent (Qiagen) into HEK293T cells. At 48 h after transfection, the luciferase activity was measured using a Dual-luciferase® reporter assay system (Promega). The data shown is derived from triplicate experiments. The ^**^ indicates significant difference (*P* < *0.01*).

The specific interaction of pmo-miR-315 with target mRNA binding site on *PmPPAE3* coding sequence was confirmed *in vitro* in HEK293T cell line. Either pmo-miR-315 mimic or its scramble miRNA and the luciferase reporter plasmid containing pmo-miR-315 binding site of *PmPPAE3* (pmir-T315) were co-transfected into HEK293T cells and then assayed for the luciferase activity. The results showed that in the presence of the mimic pmo-miR-315, the luciferase activity was reduced by about 36% compared with the reaction containing scramble miRNA (Figure 3). The reduction of luciferase activity suggested the specific binding of mimic pmo-miR-315 to miRNA-binding site of *PmPPAE3*.

### Characterization of *pmPPAE3* gene

As mentioned above, the *Pm*PPAE3 entails the shrimp immunity against WSSV. The function of PPAE is generally involved in the proPO system as reported previously by Charoensapsri et al. (22), and Charoensapsri et al. (23). Therefore, the function of *Pm*PPAE3 in the proPO system was investigated. The full-length gene of *PmPPAE3* was identified (Supplementary Figure 1). Using the 5′-RACE technique, the *PmPPAE3* full-length gene of 2,988 bp including 5′-UTR of 147 bp, 3′-UTR of 909 bp and the ORF of 1,932 bp encoding for 643 amino acid residue protein was obtained.

**Figure 4:**
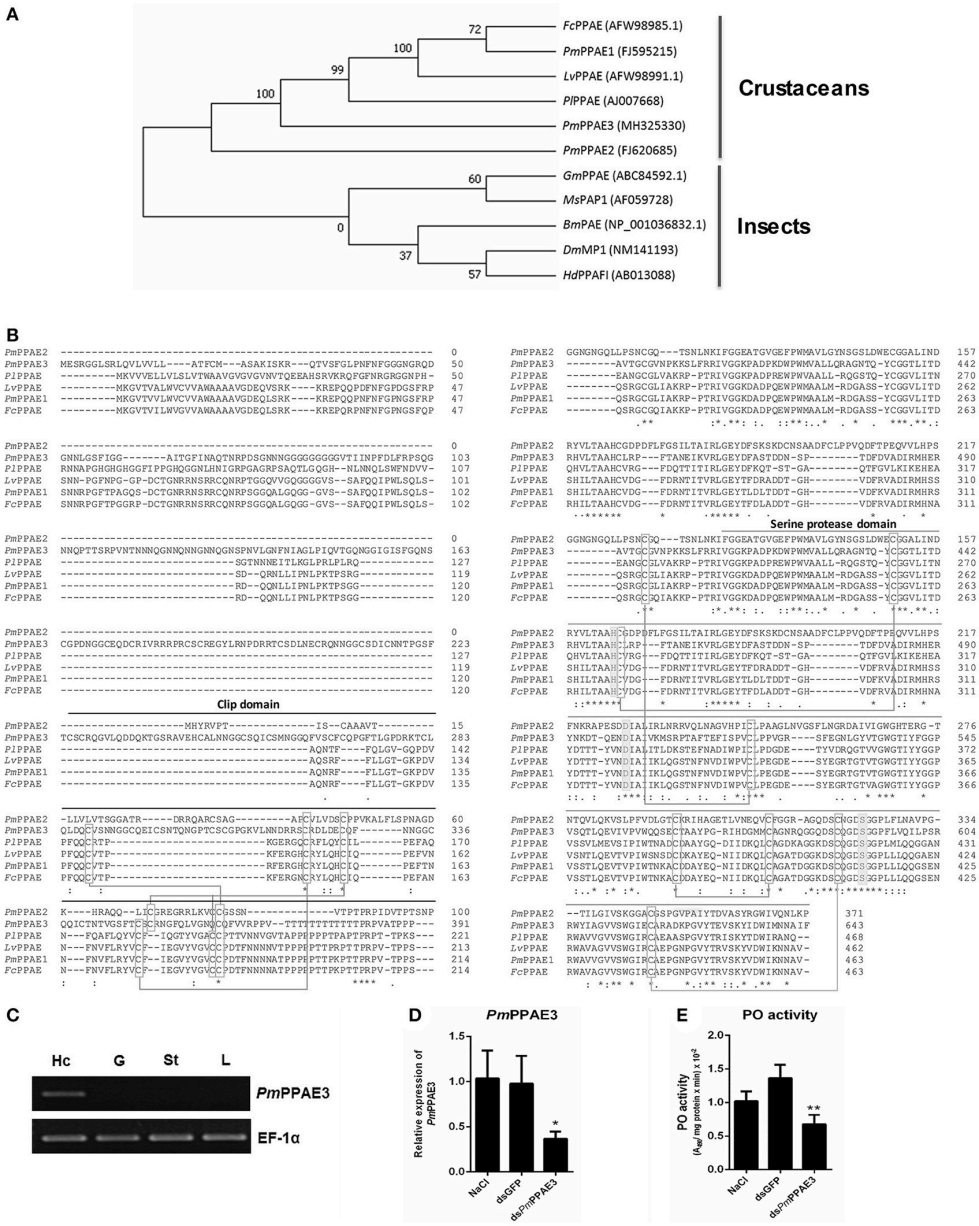
Characterization of *PmPPAE3* gene. **(A)** Phylogenetic analysis of PPAEs from various organisms including crustaceans and insects, e.g., *B. mori* PO-activating enzyme (*Bm*PAE); *M. sexta* proPO-activating proteinase 1 (*Ms*PAP1), *H. diomphalia* proPO-activating factor I (*Hd*PPAFI), *G. morsitans* (*Gm*PPAE) and *D. melanogaster* melanization protease1 (*Dm*MP1); shrimp *P. monodon* proPO-activating enzymes 1, 2 and 3 (*Pm*PPAE1, *Pm*PPAE2, *Pm*PPAE3), shrimp, *L. vannamei* proPO-activating enzyme (*Lv*PPAE), *F. chinensis* proPO-activating enzyme (*Fc*PPAE), and crayfish *P. leniusculus* proPO-activating enzyme (*Pl*PPAE), was performed using ClustaX 2.1 and MEGA7 softwares. **(B)** Multiple alignment of the deduced amino acid sequences of *Pm*PPAE3 with other crustacean PPAEs was conducted using the Clustal Omega program. The conserved features of PPAEs such as the disulfide bond, the catalytic triad (histidine, aspartic acid, and serine residues), the clip domain, and the serine proteinase domain are shown. **(C)** The representative result of *PmPPAE3* gene tissue distribution analysis by semi-quantitative RT-PCR in the hemocytes (Hc), gill (G), stomach (St), and lymphoid organ (L), is shown. In this analysis, the *EF-1*α transcript was used as an internal control. All experiments were performed in triplicate. **(D)** The *PmPPAE3* gene knockdown using *PmPPAE3*-dsRNA was performed to reveal the functional role of *Pm*PPAE in proPO activating system. The effectiveness of the *PmPPAE3* gene knockdown is shown. Hemocytes collected from the control groups of 0.85% NaCl and dsGFP injected shrimp and from the experimental shrimp injected with *PmPPAE3*-dsRNA was analyzed for the expression of *PmPPAE3* gene by qRT-PCR using the *EF-1*α transcript as an internal control. **(E)** The PO activity was assayed in the *PmPPAE3* knockdown shrimp hemocytes. The data are derived from three independently replicated experiments. The ^*^ and ^**^ indicate the significant difference at *P* < 0.05 and *P* < 0.01, respectively.

The phylogenetic tree constructed based on the deduced amino acid sequences corresponding to the ORFs of PPAEs from crustaceans and insects showed that the PPAEs are clustered into the crustacean and insect lineages. (Figure 4A). Amino acid sequence alignment of insect and crustacean PPAEs showed the conserved patterns of a clip domain, the serine proteinase domain, and the catalytic triad of histidine, aspartic acid, and serine residues (Figure 4B). Comparing with previously identified crustacean PPAEs, the *Pm*PPAE3 showed about 46.43, 46.31, and 45.98% identity with *Fc*PPAE, *Lv*PPAE and *Pm*PPAE1, respectively. Like the *Pm*PPAE1 and *Pm*PPAE2, the *Pm*PPAE3 was specifically expressed in shrimp hemocytes (Figure 4C). Lastly, the function of *Pm*PPAE3 in the proPO system was further investigated by RNA interference (Figure 4D). The *PmPPAE3* gene knockdown dramatically reduced the PO activity in shrimp hemolymph (Figure 4E) suggesting its important role in the proPO system.

### Role of pmo-miR-315 in WSSV-infected shrimp hemocytes

**Figure 5:**
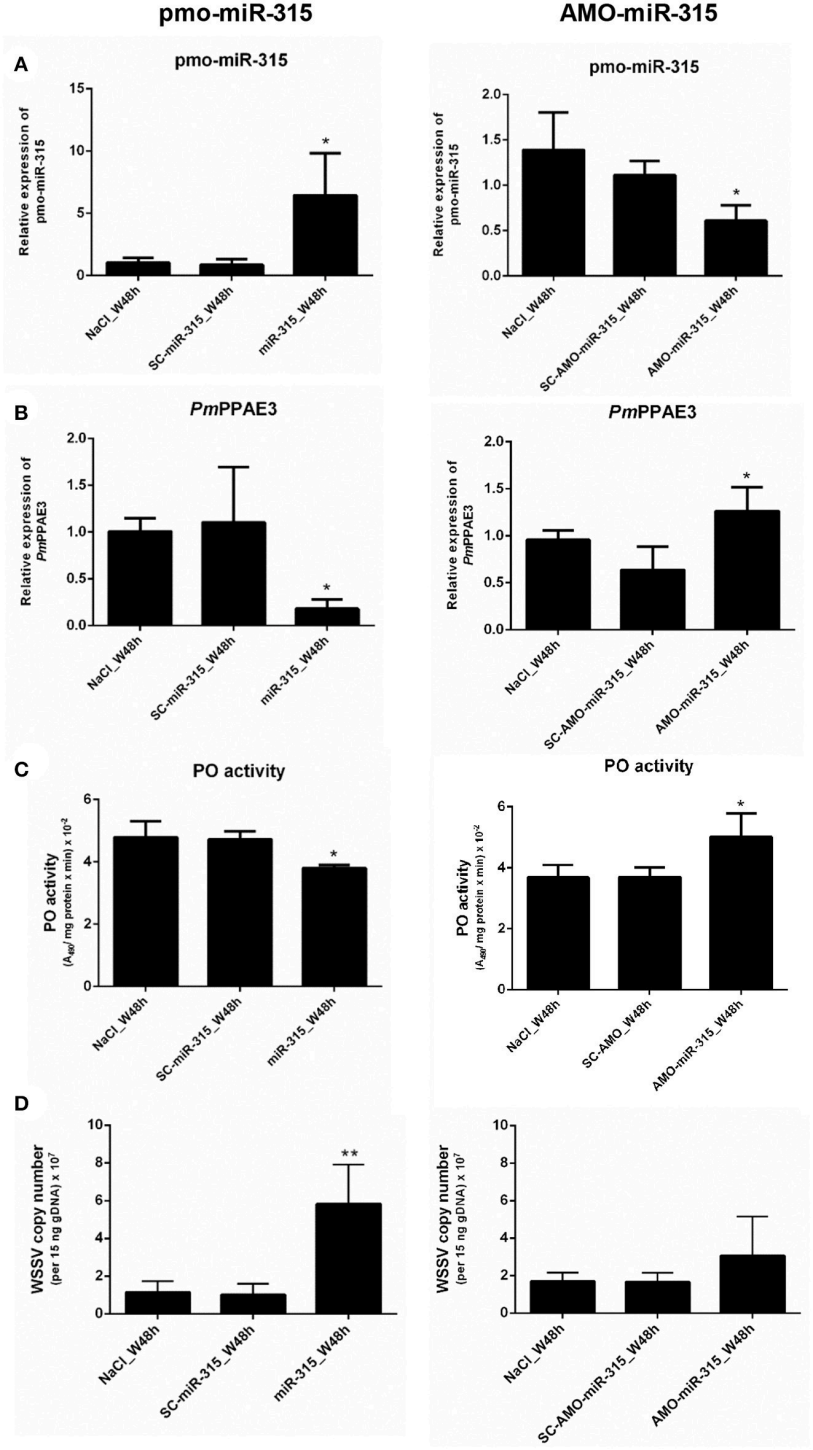
The role of pmo-miR-315 in WSSV-infected shrimp hemocytes. In this experiment, either pmo-miR-315 mimic or AMO-miR-315 was injected into WSSV-infected shrimp. Then, several parameters were investigated including **(A)** pmo-miR-315 and **(B)** *PmPPAE3* gene expression level, **(C)** PO activity and **(D)** WSSV copy number. The U6 and *EF-1*α were used as internal controls for pmo-miR-315 and *PmPPAE3* expression, respectively. All experiments were performed in triplicate. The ^*^ and ^**^ indicate the significant difference at *P* < 0.05 and *P* < 0.01, respectively.

As stated in the previous experiment, suppression of *PmPPAE3* gene expression resulted in a decrease in PO activity in shrimp hemolymph and the pmo-miR-315 could bind to specific site in the ORF of *PmPPAE3* gene. In order to reveal the role of pmo-miR-315 in WSSV-infected shrimp, overexpression of pmo-miR-315 by introducing the exogenous pmo-miR-315 mimic and the silencing of pmo-miR-315 by anti-pmo-miR-315 injection (AMO-miR-315) were performed in shrimp after WSSV infection. The expression of pmo-miR-315 and its target gene, *PmPPAE3*, was analyzed to confirm the regulatory role of pmo-miR-315 on *PmPPAE3* gene expression. As expected, the expression of *PmPPAE3* was decreased whereas that of pmo-miR-315 was highly increased (Figures 5A,B).

The PO activity was also determined in shrimp hemolymph to further elucidate the involvement of pmo-miR-315 in modulating the proPO system. The mimic pmo-miR-315 injected into the WSSV-infected shrimp resulted in a deduction of PO activity (Figure 5C, Supplementary Table S1), indicating that the pmo-miR-315 negatively regulated the shrimp proPO system. The WSSV copy number was also analyzed in hemocytes to determine the effect of introducing the pmo-miR-315 on WSSV replication in shrimp. Interestingly, as compared with those of control groups of saline and scramble miR-315 injection, the WSSV copy number was significantly increased in WSSV-challenged shrimp if the mimic pmo-miR-315 was introduced (Figure 5D). Conversely, silencing of pmo-miR-315 in WSSV-infected shrimp caused an increase in PO activity because of the up-regulation of *PmPPAE3* (Figure 5C, Supplementary Table S2). The inhibition of pmo-miR-315 activity by AMO-miR-315 slightly reduced the WSSV copy number when compared to that of pmo-miR-315 injection; however, it was still higher than the control groups (Figure 5D).

## Discussion

In 2007, the miR-315 from *Drosophila* was found to act as a potent activator of Wingless (Wg) signaling, a conserved pathway that regulates growth and tissue specification (24). Previously, the miR-315s from *M. japonicas, L. vannamei, P. monodon*, and *F. chinensis* have been identified as a viral responsive miRNA in WSSV-infected shrimp hemocytes(5, 13, 14, 25, 26) suggesting that the shrimp miR-315 might be involved in antiviral immunity. Therefore, the pmo-miR-315 function was further characterized in this research.

The expression of pmo-miR-315 was detected in various shrimp tissues including hemocytes, gill, lymphoid organ, and stomach. Interestingly, the expression level of pmo-miR-315 in hemocytes was significantly up-regulated, more than 30 times at 48 h post-WSSV infection, implying the specific role of pmo-miR-315 in shrimp antiviral response in hemocytes. Although the target gene of pmo-miR-315 was predicted as putative prophenoloxidase-activating enzyme *Pm*PPAE3 and serine/threonine protein kinase (13), in this research we focused on confirming the regulatory role of pmo-miR-315 on the expression of *PmPPAE3*.

Generally, the miRNA binds to the 3′-UTR of its target gene to regulate the translational repression and mRNA destabilization. Nevertheless, the binding site of miRNA can also be located at the 5′-UTR and coding sequence (CDS), albeit the regulatory activity may be different (27, 28). According to our prediction, the pmo-miR-315 can bind to CDS of *PmPPAE3* gene (13). The luciferase reporter assay revealed that the pmo-miR-315 mimic could interact with the specific binding site on *PmPPAE3* CDS, which was indicated by almost 36% reduction in luciferase activity.

Melanization activated by the prophenoloxidase (proPO) system is a principal innate immune response in shrimp. Upon pathogen invasion, the binding of Pattern Recognition Proteins (PRPs) to the microbial cell wall components activates the serine proteinase cascade that finally activates the final proteinases, called proPO-activating enzymes (PPAEs). The PPAEs, then, cleave the inactive proPOs to active POs leading to the initiation of melanin formation (29). In *P. monodon*, the active *Pm*PPAE1 and *Pm*PPAE2 cleaves *Pm*proPO1 and *Pm*proPO2 generating the active PO1 and PO2, respectively (30). The *Pm*PPAE3 whose mRNA is the target of pmo-miR-315 is a novel isoform of *Pm*PPAE characterized herein. It was mainly expressed in shrimp hemocytes and significantly suppressed after 24 and 48 h post-WSSV infection. As expected, the negative correlation of pmo-miR-315 expression and *PmPPAE3* gene expression was observed in hemocytes after WSSV infection.

The full-length gene of *PmPPAE3* was identified in this study and its deduced amino acid sequence was compared with other *Pm*PPAEs from various species as well as the *Pm*PPAE1 and *Pm*PPAE2 (22, 23). The multiple alignment of different PPAE amino acid sequences revealed that the *Pm*PPAE3 was closely related to *Fc*PPAE, *Lv*PPAE, and *Pm*PPAE1. The phylogenetic analysis showed that the shrimp PPAEs and a crayfish PPAE belonged to the crustaceans cluster. Besides, the *PmPPAE3* gene was hemocyte specific like *Pm*PPAE1 and *Pm*PPAE2. The function of *Pm*PPAE3 in proPO system was confirmed using the RNA interference technique. Therefore, the *Pm*PPAE3 was considered as a novel clips serine proteinase that function in the proPO system.

As mentioned above, the melanization reaction is activated through the proPO cascade after pathogen infection and unavoidably generates highly toxic molecules. These toxic intermediates are not only involved in pathogen killing but also cause the damage of the host cells (30, 31). Therefore, the activation cascade is needed to be tightly regulated. To date, several proteinase inhibitors of melanization cascade have been reported in shrimp, including the melanization inhibition protein (MIP), serine proteinase inhibitors (serpins), alpha-2-macroglobulin (A2M) and pacifastin (29). Furthermore, the small RNAs can also regulate the proPO system. The shrimp miR-100, which was up-regulated after WSSV or *V. alginolyticus* infection, can regulate several shrimp immune reactions including proPO activity, SOD activity and phagocytosis (32).

The involvement of proPO system upon WSSV infection has been reported. The PO activity is strongly decreased after WSSV-infection in shrimp (33). Later, it is found that the WSSV453 binds to and interferes with the pro*Pm*PPAE2 activation to active *Pm*PPAE2 resulting in the suppression of shrimp melanization (18, 33). In our research, the role of pmo-miR-315 and *Pm*PPAE3 in proPO system during WSSV infection in shrimp was explored. Injection of pmo-miR-315 mimic into the shrimp resulted in a reduction of *PmPPAE3* gene expression and the PO activity as expected. On the other hand, inhibition of pmo-miR-315 by AMO-miR-315 increased the *PmPPAE3* gene expression leading to the increase in PO activity in WSSV-infected shrimp hemocytes. Therefore, we can conclude that the pmo-miR-315 regulates the proPO system via *PmPPAE3* post-transcriptional repression.

Upon WSSV infection, the shrimp proPO system takes part in shrimp antiviral immunity by producing melanin and cytotoxic intermediate for viral sequestration. However, the WSSV can overcome shrimp antiviral immunity partly by proPO system suppression. Several crustacean miRNAs have been reported to promote WSSV propagation. In *M. rosenbergii*, the host miR-s9041 and miR-9850 play positive roles in WSSV replication by targeting the *STAT* gene (10). In crabs, *E. sinensis*, the miR-217 leads to a decrease in the transcript level of *Tube* gene resulting in the enhancement of WSSV copies (34). Meanwhile, the survival rate of miR-100 silenced shrimp after WSSV infection is increased as the PO activity (32). According to our research, an increase in the viral copy number in WSSV-infected shrimp after pmo-miR-315 mimic injection indicated that the pmo-miR-315 functioned in enhancing viral replication by regulating the proPO system through the inhibition of *PmPPAE3* gene expression. On the other hand, the viral copy number of AMO-miR-315 challenged shrimp was lower than that of WSSV-infected shrimp challenged with exogenous pmo-miR-315 but was not lower than the control groups as expected (Figure 5D). This might be because the pmo-miR-315 was not totally silenced. Since the whole genome sequence of *P. monodon* has not been reported yet, it was possible that the AMO-miR-315 could also target other unknown genes that might affect the WSSV propagation.

**Figure 6:**
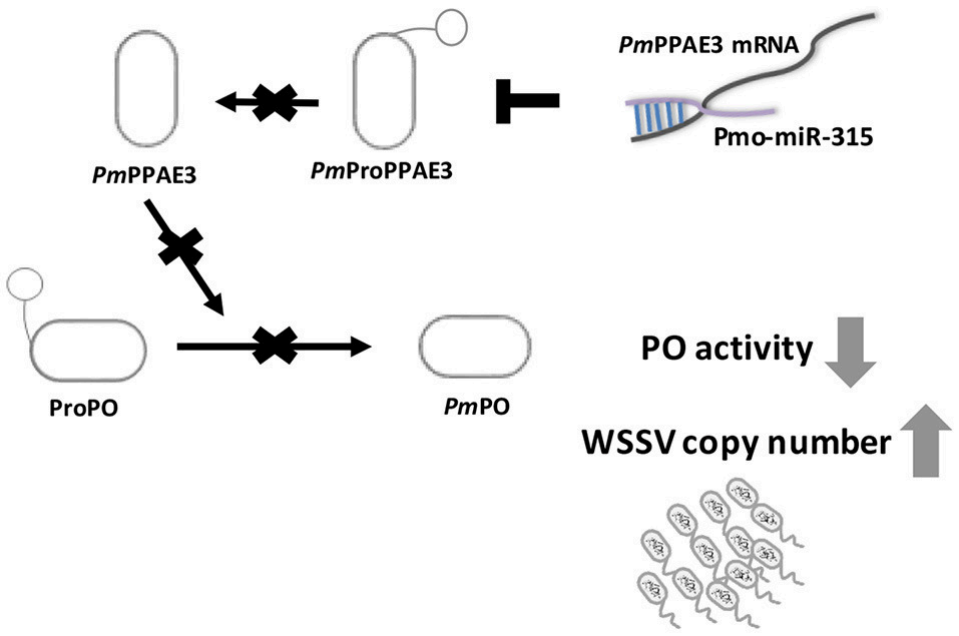
A schematic representation of the pmo-miR-315 role in modulating proPO activity by *PmPPE3* suppression during WSSV infection.

Taken together, our research offer a new mechanism on how WSSV inhibits proPO system by triggering host miRNA, as illustrated in Figure 6. The WSSV infection causes the pmo-miR-315 over-expression and subsequently inhibits the *PmPPAE3* expression leading to a decrease in PO activity and increase in WSSV copy number in WSSV-infected shrimp hemocytes. Therefore, the pmo-miR-315 regulates the proPO system through the suppression of *PPAE3* gene expression which leads to the promotion of viral propagation in shrimp hemocytes during WSSV infection.

## Author contributions

KS participated in funding acquisition and supervision of this works. KS and PJ designed and provided the resources for the study. PJ performed *in vivo* experiments. PJ and CW performed *in vitro* experiments. All the authors analyzed, investigated the data and also wrote, reviewed, and edited the manuscript.

### Conflict of interest statement

The authors declare that the research was conducted in the absence of any commercial or financial relationships that could be construed as a potential conflict of interest.
