# Supplementary material for: White Spot Syndrome Virus-Induced Shrimp miR-315 Attenuates Prophenoloxidase Activation via PPAE3 Gene Suppression
Source: Front Immunol. 2018 Sep 25;9:2184. doi: 10.3389/fimmu.2018.02184 (PMC6178132; doi:10.3389/fimmu.2018.02184)
Supplement: Supplementary file 1 [file Data_Sheet_1.PDF]

Supplementary Material

White spot syndrome virus-induced shrimp miR-315 attenuates prophenoloxidase activation via *PPAE3* gene suppression

Phattarunda Jaree[1], Chantaka Wongdontri[1], and Kunlaya Somboonwiwat[1,2]*

*** Correspondence:** Dr.Kunlya Somboonwiwat : kunlaya.s@chula.ac.th

```
   1  acatgggagttgggtccagacctccgtcagggaaccgtggcgaggagggcgtggctacac
  61  atcataatagcctaagaataaggaggaagagaaaggaattaaagaagaaggaaaaacgag
 121  gaacccacaggaagagagagacagaagatggagtcaagaggagggttaagtcgcctccag
                                        M  E  S  R  G  G  L  S  R  L  Q     11
 181  gtgcttgtggtgctgctggctacattctgcatggcttcggcgaaaatctccaagagacag
       V  L  V  V  L  L  A  T  F  C  M  A  S  A  K  I  S  K  R  Q     31
 241  actgtttcgttcgggctgcccaatttcaacttcggcggcggcaacggcaggcaggacggc
       T  V  S  F  G  L  P  N  F  N  F  G  G  G  N  G  R  Q  D  G     51
 301  aacaacctcggctccttcatcggcggagcgatcaccggcttcatcaacgcccagaccaac
       N  N  L  G  S  F  I  G  G  A  I  T  G  F  I  N  A  Q  T  N     71
 361  agacccgacagtggcaacaacaacggcggcggcggcggaggcggaggcggagtcacgatc
       R  P  D  S  G  N  N  N  G  G  G  G  G  G  G  G  G  V  T  I     91
 421  atcaacccgtttgatttattccggccgagtcaggggaataatcaaccaacgaccagtagg
       I  N  P  F  D  L  F  R  P  S  Q  G  N  N  Q  P  T  T  S  R     111
 481  cctgtgaatactaataataatcaagggaataatcaaaacaacggaaataaccaaggaaac
       P  V  N  T  N  N  N  Q  G  N  N  Q  N  N  G  N  N  Q  G  N     131
 541  agcccgaatgtccttgggaatttcaacattgccggactgcctatccaggtgactggacag
       S  P  N  V  L  G  N  F  N  I  A  G  L  P  I  Q  V  T  G  Q     151
 601  aacggaggcattggcatctccttcggacaaaacagttgcggcccggacaatggcggctgc
       N  G  G  I  G  I  S  F  G  Q  N  S  C  G  P  D  N  G  G  C     171
 661  gagcaggactgccgcatcgtccgccgaagacccaggtgttcctgccgagagggctacctg
       E  Q  D  C  R  I  V  R  R  R  P  R  C  S  C  R  E  G  Y  L     191
 721  cgcaaccccgaccgccgcacctgctccgacctgaacgagtgccgccagaacaacggcggc
       R  N  P  D  R  R  T  C  S  D  L  N  E  C  R  Q  N  N  G  G     211
 781  tgcagcgacatctgtaacaacacccccggctccttcacgtgctcttgccgccagggtgtt
       C  S  D  I  C  N  N  T  P  G  S  F  T  C  S  C  R  Q  G  V     231
 841  ctgcaggacgaccagaagaccggctcgagggcggtggagcactgcgcgctcaataacgga
       L  Q  D  D  Q  K  T  G  S  R  A  V  E  H  C  A  L  N  N  G     251
 901  gggtgttctcagatctgctccatgaacggagggcagttcgtgtcgtgcttctgccagcct
       G  C  S  Q  I  C  S  M  N  G  G  Q  F  V  S  C  F  C  Q  P     271
 961  ggcttcacgctgggacccgacaggaagacgtgcctgcagctggaccagtgtgtgagcaac
       G  F  T  L  G  P  D  R  K  T  C  L  Q  L  D  Q  C  V  S  N     291
1021  aacggaggatgtcaggagatctgcagcaacacacagaacggtcctacgtgcagctgtggg
       N  G  G  C  Q  E  I  C  S  N  T  Q  N  G  P  T  C  S  C  G     311
1081  ccgggcaaggtcctaaacaacgacaggaggagctgcagagacctggacgagtgccagttc
       P  G  K  V  L  N  N  D  R  R  S  C  R  D  L  D  E  C  Q  F     331
1141  aacaacggagggtgccagcaaatctgcaccaacactgtcggctccttcacgtgttcctgc
       N  N  G  G  C  Q  Q  I  C  T  N  T  V  G  S  F  T  C  S  C     351
1201  cgtaacgggttccaactcgtcgggaaccagtgccagttcgtcgttcgcccaccagtgacc
       R  N  G  F  Q  L  V  G  N  Q  C  Q  F  V  V  R  P  P  V  T     371
1261  accaccacaacgaccaccaccacaacgaccactcctcgcccagtggccacgccgcctcct
       T  T  T  T  T  T  T  T  T  T  T  P  R  P  V  A  T  P  P  P     391
1321  gctgtcactgggtgcggagtgaaccccaagaagtccctgttccggcgcatcgtgggggc
       A  V  T  G  C  G  V  N  P  K  K  S  L  F  R  R  I  V  G  G     411
1381  aagccggccgacccgaaggactggccgtggatggtcgctctcctgcagagggccggcaac
       K  P  A  D  P  K  D  W  P  W  M  V  A  L  L  Q  R  A  G  N     431
1441  acgcagtactgtggcgggactctcatcacagaccgccatgttctcactgctgctcactgc
       T  Q  Y  C  G  G  T  L  I  T  D  R  H  V  L  T  A  A  H  C     451
1501  ctcaggcccttcaccgccaacgagatcaaggtgcgtctcggcgagtatgacttcgcaagc
       L  R  P  F  T  A  N  E  I  K  V  R  L  G  E  Y  D  F  A  S     471
```

```
1561  acggatgacaactcgcccactgacttcgatgtggctgacatccgaatgcacgaaaggtac
       T  D  D  N  S  P  T  D  F  D  V  A  D  I  R  M  H  E  R  Y     491
1621  aacaaggacactcaggagaacgacatcgccatcgtgaagatgagtcgcccgaccgccttc
       N  K  D  T  Q  E  N  D  I  A  I  V  K  M  S  R  P  T  A  F     511
1681  accgaattcatctcgccggtctgcctgccgccggtcgggaggtccttcgagggcaacctg
       T  E  F  I  S  P  V  C  L  P  P  V  G  R  S  F  E  G  N  L     531
1741  ggctacgtcacaggctggggcaccatctacttcggcggccccgtcagccagacgctgcag
       G  Y  V  T  G  W  G  T  I  Y  F  G  G  P  V  S  Q  T  L  Q     551
1801  gaggtgatcgtgcccgtgtggcaacagagcgagtgcacggctgcgtaccctgggaggatc
       E  V  I  V  P  V  W  Q  Q  S  E  C  T  A  A  Y  P  G  R  I     571
1861  catgacggcatgatgtgcgccggcaacaggcagggcggccaggactcgtgccagggagat
       H  D  G  M  M  C  A  G  N  R  Q  G  G  Q  D  S  C  Q  G  D     591
1921  tccggaggcccattcctcgtccagatcctgccctcgcgacgctggtatatcgccggcgtg
       S  G  G  P  F  L  V  Q  I  L  P  S  R  R  W  Y  I  A  G  V     611
1981  gtatcctggggcatcgagtgtgcacgcgctgacaagcccggcgtctacacggaagtcagc
       V  S  W  G  I  E  C  A  R  A  D  K  P  G  V  Y  T  E  V  S     631
2041  aagtacattgattggataatgaacaacgccattttctaaaacgacgagtgttcgctgttg
       K  Y  I  D  W  I  M  N  N  A  I  F  -                          643
2101  cttccgcgtccgcctaactaatacacacagcccgatacttcgcgcgatcaactgtgcatt
2161  ttttatagatcaggaagaggatataaaataaagataaataatttcagaacttcgatatga
2221  aaacacatcgcaagtctaataaaagaatgcacgataatggtacttgcaatacataaaaga
2281  attaaattttataacgtttgtgggtgttattttttttctttctcacaaatgcaagtatatt
2341  agtgccgttcaaatgaaaacgaaacaagtattctgggcagataaaaaggtctgtgcaagt
2401  aatcctcagtaatagtcgacaaggaagtgatttcttgcatactgcatagatcaccactgg
2461  aattggttaagggtttcttgttacaatatgtaacacatgaaatgaaacgaagtggtgaat
2521  tccaatagatttaataggtggttttgctattacggttaatgtttacatgtgttcagttga
2581  tattatgcatatggccagtacaccagattattaatgaccactatattagagggaaagggc
2641  atttttatatttatacctgatacttaattatctaacatatcagttatatgttagtactga
2701  tatttttgtgaggatctttgttatgaaacagaccattttcggttccgtccatcaaagtgc
2761  aatcaataagataaaggcaagtatttatttcataaaaggtttctcatgagttcatatcct
2821  gcgtgatggcctcttaataatgttttcgtctttttgatttgggtatttctgagtctctct
2881  cattccatgacatcatcttattctgacatcacccagatacactgtacctaattacctttc
2941  accacattcctgaagactcttgtccaatactttgtacacttctgataa
```

Supplementary Figure 1 The full length nucleotide sequence with deduced amino acid of *Pm*PPAE3 cDNA (Accession no.: MH325330).
