# Supplementary material for: White Spot Syndrome Virus-Induced Shrimp miR-315 Attenuates Prophenoloxidase Activation via PPAE3 Gene Suppression
Source: Front Immunol. 2018 Sep 25;9:2184. doi: 10.3389/fimmu.2018.02184 (PMC6178132; doi:10.3389/fimmu.2018.02184)
Supplement: Supplementary file 2 [file Data_Sheet_2.PDF]

Supplementary Material

White spot syndrome virus-induced shrimp miR-315 attenuates prophenoloxidase activation via *PPAE3* gene suppression

Phattarunda Jaree[1], Chantaka Wongdontri[1], and Kunlaya Somboonwiwat[1,2]*

*** Correspondence:** Dr.Kunlya Somboonwiwat : kunlaya.s@chula.ac.th

Table S1: Effect of pmo-miR-315 on PO activity in WSSV-infected shrimp hemolymph

	NaCl_W48h			SC-miR-315_W48h			miR-315_W48h		
	1	2	3	1	2	3	1	2	3
A_{490} (10 min)	0.1536	0.1803	0.1343	0.1558	0.1651	0.1537	0.1312	0.1296	0.1128
Total mg protein	0.320	0.340	0.313	0.344	0.330	0.332	0.347	0.332	0.304
PO activity	0.048	0.053	0.043	0.045	0.050	0.046	0.038	0.039	0.037
**PO activity * 100**	**4.794**	**5.299**	**4.288**	**4.531**	**5.009**	**4.634**	**3.784**	**3.905**	**3.714**

Table S2: Effect of AMO-miR-315 on PO activity in WSSV-infected shrimp hemolymph

	NaCl_W48h			SC-AMO_W48h			AMO_W48h		
	1	2	3	1	2	3	1	2	3
A_{490} (10 min)	0.1234	0.1187	0.1059	0.1337	0.1139	0.1205	0.1487	0.1436	0.1901
Total mg protein	0.299	0.330	0.317	0.334	0.337	0.323	0.317	0.323	0.323
PO activity	0.041	0.036	0.033	0.040	0.034	0.037	0.047	0.044	0.059
**PO activity * 100**	**4.121**	**3.594**	**3.336**	**4.000**	**3.377**	**3.730**	**4.686**	**4.448**	**5.891**
